# Reasons for non-feasibility of therapeutic drug monitoring of oral targeted therapies in oncology – an analysis of the closed cohorts of a multicentre prospective study

**DOI:** 10.1038/s41416-024-02789-2

**Published:** 2024-07-06

**Authors:** Maud B. A. van der Kleij, Niels A. D. Guchelaar, Marinda Meertens, Kim Westerdijk, Eline L. Giraud, Roos F. Bleckman, Stefanie L. Groenland, Ruben A. G. van Eerden, Alex L. T. Imholz, Annelie J. E. Vulink, Hans-Martin Otten, Helle-Brit Fiebrich-Westra, Floor J. E. Lubberman, Ingrid M. E. Desar, Dirk-Jan A. R. Moes, Daan J. Touw, Stijn L. W. Koolen, Hans Gelderblom, An K. L. Reyners, Nielka P. van Erp, Ron H. J. Mathijssen, Alwin D. R. Huitema, Dutch Pharmacology Oncology Group (DPOG), Neeltje Steeghs

**Affiliations:** 1 Department of Clinical Pharmacology, Division of Medical Oncology, The Netherlands Cancer Institute, Antoni van Leeuwenhoek, Amsterdam, The Netherlands; 2 Department of Medical Oncology, Erasmus MC Cancer Institute, Rotterdam, The Netherlands; 3 Department of Pharmacy & Pharmacology, The Netherlands Cancer Institute, Antoni van Leeuwenhoek, Amsterdam, The Netherlands; 4 Department of Medical Oncology, Research Institute for Medical Innovation, Radboud University Medical Centre, Nijmegen, The Netherlands; 5 Department of Pharmacy and Clinical Pharmacology, Research Institute for Medical Innovation, Radboud University Medical Centre, Nijmegen, The Netherlands; 6 Department of Medical Oncology, University Medical Centre Groningen, University of Groningen, Groningen, The Netherlands; 7 Department of Medical Oncology, Deventer Hospital, Deventer, The Netherlands; 8 Department of Medical Oncology, Reinier de Graaf Hospital, Delft, The Netherlands; 9 Department of Medical Oncology, Meander Medical Centre, Amersfoort, The Netherlands; 10 Department of Medical Oncology, Isala Hospital, Zwolle, The Netherlands; 11 Department of Pharmacy, Gelderse Vallei Hospital, Ede, The Netherlands; 12 Department of Clinical Pharmacy & Toxicology, Leiden University Medical Centre, Leiden, The Netherlands; 13 Department of Clinical Pharmacy and Pharmacology, University Medical Centre Groningen, University of Groningen, Groningen, The Netherlands; 14 Department of Pharmacy, Erasmus University Medical Centre, Rotterdam, The Netherlands; 15 Department of Medical Oncology, Leiden University Medical Centre, Leiden, The Netherlands; 16 Department of Clinical Pharmacy, Utrecht University Medical Centre, Utrecht, The Netherlands; 17 Department of Pharmacology, Princess Máxima Centre for Paediatric Oncology, Utrecht, The Netherlands; 18 Department of Medical Oncology, Utrecht University Medical Centre, Utrecht University, Utrecht, The Netherlands

**Keywords:** Targeted therapies, Cancer therapy

## Abstract

**Background:**

Therapeutic drug monitoring (TDM) – performing dose adjustments based on measured drug levels and established pharmacokinetic (PK) targets – could optimise treatment with drugs that show large interpatient variability in exposure. We evaluated the feasibility of TDM for multiple oral targeted therapies. Here we report on drugs for which routine TDM is not feasible.

**Methods:**

We evaluated drug cohorts from the Dutch Pharmacology Oncology Group – TDM study. Based on PK levels taken at pre-specified time points, PK-guided interventions were performed. Feasibility of TDM was evaluated, and based on the success and practicability of TDM, cohorts could be closed.

**Results:**

For 10 out of 24 cohorts TDM was not feasible and inclusion was closed. A high incidence of adverse events resulted in closing the cabozantinib, dabrafenib/trametinib, everolimus, regorafenib and vismodegib cohort. The enzalutamide and erlotinib cohorts were closed because almost all PK levels were above target. Other, non-pharmacological reasons led to closing the palbociclib, olaparib and tamoxifen cohort.

**Conclusions:**

Although TDM could help personalising treatment for many drugs, the above-mentioned reasons can influence its feasibility, usefulness and clinical applicability. Therefore, routine TDM is not advised for cabozantinib, dabrafenib/trametinib, enzalutamide, erlotinib, everolimus, regorafenib and vismodegib. Nonetheless, TDM remains valuable for individual clinical decisions.

## Background

Currently available information on pharmacokinetic (PK) and pharmacodynamic (PD) data suggests that oral targeted therapies usually demonstrate large interpatient variability in drug exposure [1]. As patients are often treated with a fixed standard dose, this could lead to unnecessary underdosing and overdosing of patients, potentially resulting in reduced efficacy and avoidable toxicity. One approach to personalise treatment with oral targeted therapies could be through therapeutic drug monitoring (TDM). TDM consists of measuring PK levels and adjusting the dose based on these PK levels and pre-specified targets (PK-guided interventions). There are several drug specific criteria that make drugs suitable TDM candidates. Alongside considerable interpatient variability in exposure, a reliable bio-analytical method for measuring PK levels should be available, drugs should have a narrow therapeutic window and there should be no superior biomarker for assessing drug effect. Additionally, therapy should be long ongoing, so there is time for dose adjustments, and there should be feasible dose adjustment options, which is often so when drugs have dose-proportional pharmacokinetics. Lastly, TDM is mostly useful when an exposure-response relationship with an accompanying target has been established. Many oral targeted therapies fit these criteria [2].

In oncological drug development, dose selection has traditionally been based primarily on determining the maximum tolerated dose (MTD) [3]. For the increasingly more available oral targeted therapies, dosing at the MTD might not necessarily enhance efficacy because of target saturation, and the optimal biological dose is often lower than the MTD [4, 5]. Consequently, the Food & Drug Administration proposed Project Optimus, an initiative to revise dose selection of anticancer drugs, guided by PK/PD data instead of toxicity [4, 5]. Because these PK/PD data were previously often not available during the dose finding phase, dose- and exposure-response data for many currently registered oral targeted therapies were initially lacking. In recent years, further studies have delineated the existence of exposure-response relationships at the standard dose for these drugs [6], and Project Optimus is now contributing to an increased availability of PK/PD data of newly developed drugs [5].

Given the enhanced availability of PK/PD data, multiple studies have demonstrated the feasibility of the use of routine TDM for obtaining adequate PK levels for multiple oral targeted therapies, such as abiraterone, tamoxifen, imatinib, pazopanib and sunitinib [6–15]. We have investigated the feasibility of TDM for over twenty drugs, and demonstrated that overall TDM is feasible, however this feasibility was not clear for all separate drugs [16]. For the VEGFR-inhibitor (vascular endothelial growth factor receptor inhibitor) sorafenib for example, we showed that TDM was not feasible due to high incidence of treatment related adverse events (trAEs) [17].

The aim of the current study was to report why TDM was not feasible or applicable for some of the oral targeted therapies. With our findings, we aim to provide recommendations for overcoming these challenges and suggest potential directions for future research on feasibility of TDM in oncology.

## Methods

### DPOG-TDM study

**Fig. 1:**
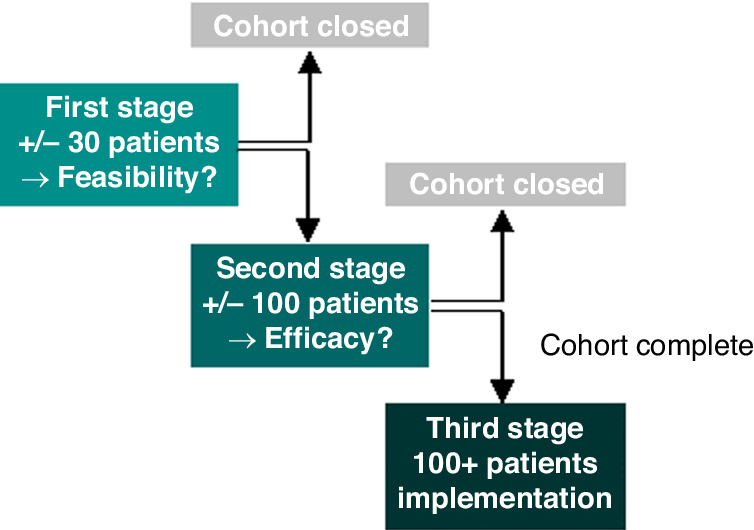
Overview of stages. The study included a maximum of three stages per drug cohort. After the first stage, feasibility was evaluated. If considered promising, the cohort proceeded to the second stage, to confirm feasibility in a larger cohort and to evaluate preliminary efficacy. After evaluation, the cohorts were either closed or continued to the implementation stage.

This analysis is part of a larger project: the Dutch Pharmacology Oncology Group (DPOG)-TDM study (https://trialsearch.who.int/, trial number NTR6866). This is a dynamic prospective study in twelve hospitals in the Netherlands. This study was performed on behalf of the DPOG, a consortium consisting of hospital pharmacists and medical oncologists from five Dutch university hospitals/ tertiary care cancer centres (Erasmus MC Cancer Institute, Leiden University Medical Centre, Netherlands Cancer Institute-Antoni van Leeuwenhoek (NKI-AVL), Radboud University Medical Centre and University Medical Centre Groningen) performing research on clinical pharmacology in oncology. Patients starting with one of the included oral targeted therapies on the standard dose could be enrolled. The protocol has been published elsewhere [18]. The DPOG-TDM study was approved by the Institutional Research Board of the NKI-AVL and by each individual participating hospital. All patients provided written informed consent. Patients were included between June 2017 and October 2022. The study included a maximum of three stages per drug cohort (Fig. 1). The aim of the first stage was to evaluate the feasibility of PK-guided interventions per cohort. These evaluations were done when approximately 30 patients were included within each cohort, or sooner if indicated. Whenever the feasibility of PK-guided interventions was considered promising, the cohort proceeded to the second stage, with the aim to confirm feasibility in a larger cohort (*n* = 100) and to evaluate preliminary efficacy results. After evaluation, the cohort was either closed or continued into the implementation phase (third stage). Feasibility of TDM was evaluated based on possibility to perform PK-guided interventions and their successfulness. PK-guided interventions were considered successful if the median C_min_ after the intervention was above target and if no dose limiting trAEs were observed within the first month following intervention.

### Patient selection

Patients from cohorts which were closed during or after either the first or second stage were included in this analysis (as per January 2024). This includes patients from the cabozantinib, dabrafenib/trametinib, enzalutamide, erlotinib, everolimus, olaparib, palbociclib, regorafenib, tamoxifen and vismodegib cohort. The lack of feasibility of TDM of the sorafenib cohort has been discussed elsewhere, so patients from the sorafenib cohort were therefore not included in this analysis [17]. Collected data included patient and tumour characteristics, PK levels, recommended and performed PK-guided interventions and reasons if not performed, treatment outcomes, details on treatment discontinuation and survival and clinically relevant trAEs (according to the Common Terminology Criteria for Adverse Events (CTCAE) grading system). Clinically relevant trAEs were defined as AEs ultimately leading to treatment discontinuation, dose reductions, treatment interruptions or restricting dose interventions.

### PK-guided interventions

PK levels were taken at four, eight and twelve weeks after start of treatment, and every twelve weeks thereafter. Because of their long elimination half-lives, tamoxifen and vismodegib PK levels were drawn every twelve weeks from start of treatment; for palbociclib PK levels were taken at three, seven and eleven weeks because of the three weeks on, one week off dosing scheme. Date and time of the last drug ingestion was noted. PK levels were measured using validated assays [19]. PK levels were drawn either pre-dose (minimal plasma concentration, C_min_ level), or at random time after dose, but after the time to maximum exposure (T_max_). If drawn at random time after dose, PK levels were extrapolated to a C_min_ using log-linear extrapolation [20, 21]. For tamoxifen, there was no need for extrapolation because of the long half-life of the active metabolite endoxifen, where the target was based on. For olaparib, we used the ratio of the average population concentration and the measured concentration multiplied with the average population value C_min_ to predict the C_min_, as it follows multicompartmental kinetics [20–22]. If there was a PK level below target (specified in Table 1), a PK-guided intervention was recommended. Possible PK-guided interventions were specific per drug and included dose escalation, emphasis on drug compliance, and adjustment of interacting medication [18]. PK-guided interventions were only carried out if both the physician and patient agreed with the intervention and if deemed feasible (based on i.e. tolerability). In the dabrafenib/trametinib cohort PK-guided interventions were recommended based on the trametinib levels due to the exposure-response relationship for trametinib and the lack of an exposure-response relationship for dabrafenib at the approved dose [6]. For cabozantinib the standard dose in our study was 40 mg once daily (OD), and the target was based on this dose [23].

**Table 1:** Targets used for Therapeutic Drug Monitoring.

Drug	TDM target (ng/mL unless otherwise stated)	Based on
Cabozantinib	≥ 750	Average exposure of 40 mg OD at steady state [23]
Dabrafenib/ trametinib	≥ 10.6	Cut-off value from exposure-response analysis based on mean C_min_ of standard dose at steady state from previous research [41, 42]
Enzalutamide	≥ 5 mg/L	Mean C_min_ of lowest dose at steady state in phase 1-2 study [35]
Erlotinib	≥ 500	Preclinical results on EGFR inhibition [24]
Everolimus	≥ 10	Cut-off value from exposure-response analysis [43]
Olaparib	≥ 1290	Mean C_min_ of 400 mg BID capsule formulation at steady state [44]
Palbociclib	≥ 61	Mean C_min_ of standard dose at steady state [45]
Regorafenib	≥ 1400	Mean C_min_ of standard dose [46]
Tamoxifen	≥ 5.97	Threshold for at risk subgroup, corresponding closely to lowest quantile in study sample [47]
Vismodegib	≥ 11.4	Mean C_min_ of standard dose at steady state [48]

Targets are all C_min_, except tamoxifen: steady state concentration of the active metabolite endoxifen. Target for dabrafenib/trametinib cohort is based on trametinib exposure. *TDM* Therapeutic drug monitoring; *OD* Once daily, *C*_*min*_ Minimum plasma concentration, *EGFR* Epidermal growth factor receptor, *BID* Twice daily.

### Statistics

As this is a descriptive analysis, no formal hypothesis testing was performed. PK levels were analysed and extrapolated using R, version 4.2.2 (R Project, Vienna, Austria). Median exposure was calculated by calculating the median exposure for every individual patient, and ultimately calculating the median of these medians, as this result was least influenced by outliers.

## Results

As per January 1st 2024, 24 drug cohorts have been opened in the DPOG-TDM study, of which 16 cohorts have been evaluated for stage progression. Next to the sorafenib cohort, 10 other cohorts were closed after evaluation.

### Patient characteristics

**Fig. 2:**
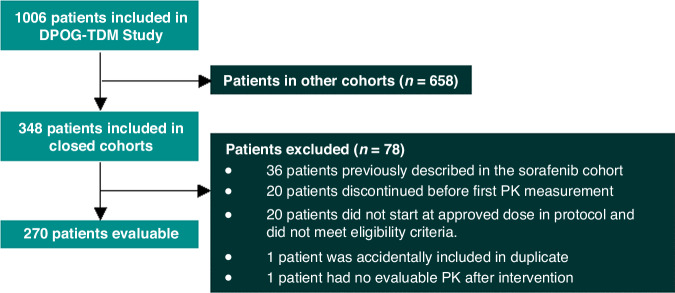
Flow chart for patient selection. *N* = number of patients. In the whole DPOG-TDM study, 1006 patients were selected. Patients in still open cohorts were not evaluable for this analysis. For the remaining patients in the closed cohorts, other reasons for exclusion are described.

In these 10 closed cohorts, 270 patients were evaluable for analysis (Fig. 2). Baseline characteristics of these patients per cohort are described in Table 2. The largest cohort was the dabrafenib/trametinib cohort, which included 65 evaluable patients, followed by enzalutamide (*n* = 42), palbociclib (*n* = 32), olaparib (*n* = 35), everolimus (*n* = 25), cabozantinib (n = 25), tamoxifen (*n* = 22), vismodegib (*n* = 12), regorafenib (*n* = 9) and erlotinib (*n* = 3).

**Table 2:** Baseline characteristics.

	All patients	Cabozantinib	Dabrafenib/ trametinib	Enzalutamide	Erlotinib	Everolimus	Olaparib	Palbociclib	Regorafenib	Tamoxifen	Vismodegib
**Total patients**	**270**	25	65	42	3	25	35	32	9	22	12
**Age at baseline** (years)	**63 [54–71]**	64 [58–71]	56 [51–65]	71.5 [68–75]	58 [51-66.5]	63 [56–69]	63 [57.5-70]	56.5 [52–64]	57 [48–73]	55.5 [48–69]	71 [62–74]
**Gender**											
Male	**119 (44.1%)**	15 (60%)	36 (55.4%)	42 (100%)	1 (33.3%)	9 (36%)	0 (0%)	0 (0%)	9 (100%)	1 (4.5%)	6 (50%)
Female	**151 (55.9%)**	10 (40%)	29 (44.6%)	0 (0%)	2 (66.7%)	16 (64%)	35 (100%)	32 (100%)	0 (0%)	21 (95.5%)	6 (50%)
**Tumour type**											
Basal cell carcinoma	**12 (4.4%)**										12 (100%)
Breast cancer	**64 (23.7%)**					10 (40%)		32 (100%)		22 (100%)	
Gastro intestinal stromal tumour	**7 (2.6%)**								7 (77.8%)		
Hepatocellular carcinoma	**2 (0.7%)**								2 (22.2%)		
Melanoma	**65 (24.1%)**		65 (100%)								
Neuroendocrine tumour	**11 (4.1%)**					11 (44%)					
Non-small-cell lung cancer	**3 (1.1%)**				3 (100%)						
Ovarian cancer	**35 (13%)**						35 (100%)				
Prostate cancer	**42 (15.6%)**			42 (100%)							
Renal Cell carcinoma	**27 (10%)**	25 (100%)				2 (8%)					
Soft-tissue sarcoma	**1 (0.4%)**					1 (4%)					
Other	**1 (0.4%)**					1 (4%)					
**Number of previous lines of systemic treatment**											
0	**103 (38.1%)**	7 (28%)	33 (50.8%)	26 (61.9%)	1 (33.3%)	5 (20%)	1 (2.85%)	5 (15.6%)		16 (72.7%)	9 (75%)
1	**81 (30.1%)**	7 (28%)	25 (38.5%)	7 (16.7%)	1 (33.3%)	5 (20%)	17 (48.6%)	9 (28.1%)	2 (22.2%)	5 (22.7%)	3 (25%)
2	**58 (21.5%)**	9 (36%)	7 (10.8%)	5 (11.9%)	1 (33.3%)	7 (28%)	13 (37.1%)	10 (31.3%)	5 (55.6%)	1 (4.6%)	
3	**18 (6.7%)**	1 (4%)		2 (4.75%)		6 (24%)	3 (8.6%)	5 (15.6%)	1 (11.1%)		
≥ 4	**10 (3.7%)**	1 (4%)		2 (4.75%)		2 (8%)	1 (2.85%)	3 (9.4%)	1 (11.1%)		
**PK levels total**	**1338**	111	324	240	13	88	232	129	30	138	33
**PK levels per patient**	**3** [2–6]	3 [2–6]	3 [2–6]	5 [3–7]	3 [2.5–5.5]	3 [1–4]	6 [3-9.5]	3 [2–5]	2 [1–3]	3 [2–9]	1.5 [1–3]
**Median C**_**min**_ **(ng/mL, unless otherwise stated)**		696 [629–897]	15.7 [13.2–19.5]	11.0 mg/L [10.0–12.8]	1543 [1319-1874]	11.1 [9.40–17.0]	1507 [1155–2243]	60.1 [46.8–75.0]	1330 [867.6–1600]	9.77 [7.11–12.0]	8.75 [7.28–11.2]

Data are expressed as number (%) or median [IQR]. Due to rounding, total percentages could be deviating from 100%. The bold values are the values for all patients combined. *PK* Pharmacokinetic, *C*_*min*_ Minimum plasma concentration

### PK levels and PK-guided interventions

In total, 1338 PK levels were drawn, with a median of three per patient [Interquartile range (IQR) 2-6]. Almost half of the patients (47%) had at least one PK level below target. An overview of PK levels and interventions can be found in Table 3.

**Table 3:** Overview of interventions per drug.

	All patients																		Main reason for closing the cohort
		≥ 1 low C_min_												All adequate PK levels					
			Intervention					No intervention							Treatment continued	Dose reduction due to toxicity	Treatment discontinued due to toxicity	Treatment interruption due to toxicity	
				Successful	Not successful				Toxicity	Physician adherence	Discon- tinued	Logistics	Borderline low PK levels						
						Still low PK levels	Toxicity												
All drugs	270	127 (47)	47 (37)	30 (63.8)	17 (26.2)	9 (52.9)	8 (47.1)	80 (63)	46 (57.5)	15 (18.8)	8 (10)	6 (7.5)	5 (6.3)	143 (53)	103 (72)	16 (11.2)	12 (8.4)	12 (8.4)	
Cabozantinib	25	20 (80)	5 (25)	2 (40)	3 (60)	1 (33.3)	2 (66.7)	15 (75)	14 (93.3)	0 (0)	0 (0)	1 (6.7)	0 (0)	5 (20)	4 (80)	0 (0)	1 (20)	0 (0)	Toxicity
Dabrafenib/ trametinib	65	18 (27.7)	6 (33.3)	4 (66.7)	2 (33.3)	1 (50)	1 (50)	12 (66.7)	3 (25)	3 (25)	3 (25)	1 (8.3)	2 (16.7)	47 (72.3)	33 (70.2)	2 (4.3)	0 (0)	12 (25.5)	Toxicity
Enzalutamide	42	2 (4.8)	1 (50)	1 (100)	0 (0)	0 (0)	0 (0)	1 (50)	0 (0)	0 (0)	1 (100)	0 (0)	0 (0)	40 (95.2)	32 (80)	6 (15)	2 (5)	0 (0)	PK levels above target
Erlotinib	3	0 (0)	0 (0)	0 (0)	0 (0)	0 (0)	0 (0)	0 (0)	0 (0)	0 (0)	0 (0)	0 (0)	0 (0)	3 (100)	3 (100)	0 (0)	0 (0)	0 (0)	PK levels above target
Everolimus	25	13 (52)	4 (30.8)	2 (50)	2 (50)	1 (50)	1 (50)	9 (69.2)	7 (77.8)	0 (0)	0 (0)	1 (11.1)	1 (11.1)	12 (48)	2 (16.7)	3 (25)	7 (58.3)	0 (0)	Toxicity
Olaparib	35	27 (77.1)	17 (63)	12 (70.6)	5 (29.4)	4 (80)	1 (20)	10 (37)	5 (50)	1 (10)	1 (10)	2 (20)	1 (10)	8 (22.9)	6 (75)	2 (25)	0 (0)	0 (0)	Other
Palbociclib	32	22 (68.7)	7 (31.8)	4 (57.1)	3 (42.9)	1 (33.3)	2 (66.7)	15 (68.2)	9 (60)	4 (26.7)	0 (0)	1 (6.7)	1 (6.7)	10 (31.3)	8 (80)	2 (20)	0 (0)	0 (0)	Other
Regorafenib	9	6 (66.7)	1 (16.7)	0 (0)	1 (100)	1 (100)	0 (0)	5 (83.3)	1 (20)	3 (60)	1 (20)	0 (0)	0 (0)	3 (33.3)	2 (66.7)	1 (33.3)	0 (0)	0 (0)	Toxicity
Tamoxifen	22	7 (31.8)	5 (71.4)	4 (80)	1 (20)	0 (0)	1 (100)	2 (28.6)	2 (100)	0 (0)	0 (0)	0 (0)	0 (0)	15 (68.2)	13 (86.7)	0 (0)	2 (13.3)	0 (0)	Other
Vismodegib	12	12 (100)	1 (8.3)	1 (100)	0 (0)	0 (0)	0 (0)	11 (91.7)	5 (45.5)	4 (36.4)	2 (18.2)	0 (0)	0 (0)	0 (0)	0 (0)	0 (0)	0 (0)	0 (0)	Toxicity

Data are expressed as number (%). *Due to rounding, total percentages could be deviating from 100%*. C_min_: minimum plasma concentration; *PK* Pharmacokinetic.

Patients had ≥ 1 low C_min_ or all adequate PK levels. If patients had ≥ 1 low C_min_ they did or did not have an intervention. If they had an intervention, it was described as successful when median C_min_ after intervention was above target and there was no dose-limiting toxicity within one month, or otherwise as a not successful intervention. If not a successful intervention, it was stated why. If no intervention was performed, the reason why was also stated. If patients had all adequate PK levels, it was stated if patients continued with the standard dose or if toxicity led to dose reduction, treatment discontinuation or treatment interruption. Lastly, the main reason for closing the cohort was stated.

### Reasons for closing cohorts

Cohorts were closed for two pharmacological reasons: 1. High incidence of toxicity (five cohorts) and 2. Almost all PK levels were above the predefined targets (two cohorts). Three cohorts were closed for other, non-pharmacological reasons.

#### Pharmacological reasons

##### Toxicity

The most common reason for closing a cohort was a high incidence of clinically relevant trAEs, whereby TDM following our protocol was not feasible.

For **cabozantinib**, 25 patients had a total of 111 available PK levels. Median exposure was 696 ng/mL and 20 patients had ≥1 PK level below target of 750 ng/mL. PK-guided interventions were only possible in five patients. Of these, the intervention was successful in two patients. Out of the three patients in which the intervention was not successful, two had to swiftly switch back to standard dose because of trAEs at the higher dose and one still had low PK levels. Of the remaining 15 patients with a PK level below target, 14 did not have an intervention because of trAEs at the standard dose. Out of all 25 patients, 19 experienced clinically relevant trAEs throughout the study, of which one started experiencing toxicity after an intervention. Many patients experienced multiple trAEs, with diarrhoea (*n* = 8), hand-foot syndrome (HFS) (*n* = 6) and fatigue (*n* = 4) being most common. Five patients had grade ≥3 trAE (mostly diarrhoea, *n* = 3). Because PK-guided interventions were often not possible due to the high trAE incidence, TDM for cabozantinib following our protocol was determined not feasible and the cohort was closed.

In the **dabrafenib/trametinib** cohort, 324 PK levels were available from 65 patients with a median trametinib exposure of 15.7 ng/mL. Eighteen patients had ≥ 1 PK level below target (trametinib C_min_ of 10.6 ng/mL). An intervention was recommended in six patients, which was successful in four. Reasons not to perform an intervention in the other 12 patients were various, with the main reasons being toxicity (*n* = 3) and physician adherence (*n* = 3). Of all 65 patients, 47 patients had all adequate PK, of which 33 patients continued treatment and the other 14 patients experienced clinically relevant trAEs. In total, 22 patients experienced clinically relevant trAEs during the study, and most often this was fever (*n* = 14). TDM was considered not feasible because of this toxicity incidence, and the cohort was closed.

In the **everolimus** cohort, 25 patients had 88 available PK levels, with a median C_min_ of 11.1 ng/mL. Out of 25 patients, 13 patients had ≥ 1 PK level below target (C_min_ of 10 ng/mL). An intervention was possible in four patients, of which two were successful. In nine patients it was impossible to perform PK-guided interventions, mostly because of toxicity (*n* = 7). Out of all 25 patients, 21 patients experienced clinically relevant trAEs, of which many of them experienced more than one. Most frequent trAEs included fatigue (*n* = 6), pneumonitis (*n* = 4), nausea/vomiting (*n* = 3) and diarrhoea (*n* = 3). Five patients experienced grade ≥ 3 trAEs. This noticeably high toxicity incidence was why the cohort was closed.

For **regorafenib**, nine patients starting on the standard dose had a total of 30 available PK levels with a median C_min_ of 1330 ng/mL. Six patients had ≥ 1 PK level below target (C_min_ of 1400 ng/mL), and an intervention was possible in only one patient, which was unsuccessful. In the five patients without an intervention, this was most often due to physician adherence (*n* = 3). In total, six patients experienced clinically relevant trAEs, of which two patients experienced HFS grade 3. Remarkably,﻿ 10 patients were enrolled while not fulfilling eligibility criteria and thus not taken into account in the feasibility analysis. In detail, all started on a lower dose of 80 mg OD (*n* = 4) or 120 mg OD (*n* = 6). From these 10 patients, 46 PK levels were measured, with a median C_min_ of 2095 ng/mL. Six patients had all adequate PK and four patients had a PK level below target (although based on the mean C_min_ of the approved dose of 160 mg OD). An intervention was possible in one patient, although unsuccessful due to toxicity. Toxicity was also the reason the other three patients with low PK levels could not undergo dose escalation. Out of the 10 patients, eight experienced clinically relevant trAEs. The high incidence of toxicity in both the standard-dose and lower-dose group made us decide to close the cohort, because TDM was deemed not feasible.

For **vismodegib**, 12 patients had a total of 33 PK levels available. All patients had at least one PK level below target (C_min_ of 11.4 ng/mL), with a median C_min_ of 8.75 ng/mL per patient. Dose escalation was successful in one patient, although later in treatment this dose was reduced again because of trAEs. The other 11 patients did not undergo a PK-guided intervention, because of trAEs (*n* = 5), physician adherence (*n* = 4) or because treatment was discontinued before dose-escalation was possible (*n* = 2). Half of the patients experienced clinically relevant trAEs, most often myalgia (*n* = 3). Because of this high level of toxicity, TDM was assumed not feasible, and the cohort was closed.

##### Almost all PK levels above the predefined target

The cohorts of enzalutamide and erlotinib were closed because almost all PK levels were (already) above the predefined target and therefore it was determined that PK-guided interventions were not of added value with the set target.

In the **enzalutamide** cohort, 42 patients were included with a total of 240 PK levels. Median C_min_ was 11.0 mg/L. Only two patients had a PK-level below target (C_min_ of 5 mg/L). A PK-guided intervention was successful in one of these patients, the other patient discontinued treatment before dose escalation was possible. Because almost all patients had PK levels above target, combined with the lacking evidence of an exposure-response relationship [6], we decided that TDM following our protocol was not of additional value, and the cohort was closed.

For the **erlotinib** cohort, the target of 500 ng/mL was based on preclinical data [6, 24]. After including three patients, with 13 PK levels, it became clear that this target was a three-fold lower than the median concentration (median C_min_ of 1543 ng/mL). All patients had adequate PK and continued with the standard dose. The absence of proof of an exposure-response relationship, combined with additional research demonstrating that 500 ng/mL is far below the median C_min_ [25, 26], and our results that all patients had far higher exposure than the target, made us decide to close the cohort.

#### Non-pharmacological reasons

The olaparib, palbociclib and tamoxifen cohorts were closed because of other, non-pharmacological reasons.

In the **olaparib** cohort, 35 patients had a total of 232 PK levels, with a median C_min_ of 1507 ng/mL and a target of 1290 ng/mL. Out of 35 patients, 27 patients had ≥1 PK level below target. An intervention was possible in 17 patients, of which 12 were successful. For 5 out of 10 patients for whom an intervention was not possible, this was because of toxicity. Although primary feasibility results were positive, a logistical issue occurred. The set target was based on the average mean C_min_ of the - no longer approved - capsule formulation, and evidence showed different exposure levels for the currently used tablet formulation [27]. Combined with the lacking evidence of an exposure-response relationship at the approved dose, we decided to close the cohort [6, 28].

In the **palbociclib** cohort 32 patients were included, with a median C_min_ of 60.1 ng/mL over 129 PK levels, of which 22 had ≥ 1 PK level below target (C_min_ of 61 ng/mL). Of these, seven had an intervention, of which four were successful. No intervention was carried out in 15 patients, primarily because of toxicity (*n* = 9). During the study, it became clear that the protocol was often not followed (59% of patients). It was impractical for patients to visit the hospital in two consecutive weeks, because of the need for C_min_ level measurements in week three of therapy and evaluation of toxicity in the rest week (week four). Taken together with the shortcoming evidence for an exposure-response relationship at the approved dose [6], we decided that TDM for palbociclib following our study protocol was not feasible in clinical practice.

In the **tamoxifen** cohort, 22 included patients with a total of 138 PK levels had a median C_min_ of 9.77 ng/mL, with a target of 5.97 ng/mL. Out of these 22 patients, seven patients had 1≥ low PK level, and an intervention was possible in five patients, of which four were successful. The other 15 patients had all adequate PK. During our study, other studies demonstrated good feasibility of TDM of tamoxifen in a far larger groups (*n* = 122 and *n* = 145, respectively) [9, 10]. Because we would not be able to meet the power of these studies, we decided that continuing inclusions in this cohort would not be of added value.

## Discussion

In this study, we aimed to report why routine TDM was not feasible for some oral targeted therapies in a multicentre prospective study. From 10 different drug cohorts with 270 patients, we closed seven cohorts because of two pharmacological reasons: high toxicity incidence and because almost all PK levels above target, and three cohorts because of other, non-pharmacological reasons. Although certain drugs might seem suitable candidates for TDM on forehand, the course of our study showed that there are several factors to consider when determining the feasibility of TDM and the applicability in clinical practice.

We closed most cohorts because of non-feasibility due to high toxicity incidence. For drugs with lacking evidence for an exposure-response relationship at the standard dose, such as vismodegib but also sorafenib [17], the decision to close the cohort was clear, as following TDM could put the patients at risk for serious toxicity without additional benefit. For oral targeted therapies with possible or inconclusive evidence for an exposure-response relationship at the standard dose – i.e. cabozantinib, everolimus, trametinib and regorafenib [6] - the non-feasibility due to toxicity raised the question whether the used targets were too high to be achievable - as toxicity was often present in patients with exposure lower than the target -, whether the therapeutic window of these drug was too small for TDM, or whether the standard dosing was too high to begin with. For example for cabozantinib, the 750 ng/mL target was based on the average exposure of the 40 mg dose at steady state derived from a population PK model. Not only did 20 out of 25 patients have ≥ 1 PK level below target, median exposure of all patients was below this target as well (696 ng/mL), with only 32% of patients having a median C_min_ above target. Results from two other trials, including patients using mostly 40 mg or 60 mg OD also showed a median exposure below 750 ng/mL (*n* = 59, C_min_ = 572 ng/mL; *n* = 76, C_min_ = 500 ng/mL) [29, 30], suggesting that the cabozantinib target in our study was too high to be feasible. For the cohorts that where closed due to toxicity, titrating the dose based on toxicity could be a useful alternative to TDM. This is already done for axitinib, a VEGFR-inhibitor (like cabozantinib and regorafenib) used in patients with renal cell carcinoma. A significantly higher proportion of patients taking axitinib following dose titration based on treatment induced hypertension achieved an objective response rate than patients following standard dosing. This was supported by exposure data, as patients who were not eligible for dose titration already therapeutic exposure on the standard dose [31–33]. In clinical practice many patients already start with cabozantinib at 40 mg OD and not the approved 60 mg OD, and are titrated to higher dosages when possible. So, dose titration supported by exposure information could be a way of getting patients at their individual MTD with most optimal treatment outcomes. Furthermore, finding better PD biomarkers is urgently needed to enable to give the patient a dose with optimal efficacy and no unnecessary toxicity and could help personalising treatment.

For the two cohorts that were closed because almost all patients had PK levels above the predefined target, the targets were based on available evidence on target saturation. For erlotinib, preclinical pharmacodynamic studies in animals suggest that concentrations above 500 ng/mL provide sufficient and almost complete EGFR (epidarmal growth factor receptor) inhibition [24]. In the phase 1 trial, most patients achieved this concentration when treated with at least 150 mg OD. The mean C_min_ of both patients receiving 150 mg OD and 100 mg OD were far above this 500 ng/mL target (1200 ng/mL and 820 ng/mL, respectively). Following the MTD strategy, 150 mg OD was advised as standard dose, but it was also stated that the effects of the dose and concentration on EGFR inhibition should be further elucidated [24]. Overall, there are indications that the standard dose of 150 mg OD is higher than needed for target saturation. For enzalutamide, the phase 1-2 trial advised 160 mg OD as standard dose, following the MTD strategy [34, 35]. It was suggested that androgen receptor binding was higher in patients treated with 150 mg OD with a C_min_ of 11.4 mg/L than in patients treated with 60 mg OD with a C_min_ of 5.0 mg/L, following positron emission tomography scans, but this difference was only minimal [35, 36]. This could indicate that there is already target saturation at C_min_ levels of > 5.0 mg/L, and this could be reached with a lower dose than 150 mg OD, for example (based on available tablets) with 120 mg OD. This is also suggested by a 2024 study on 120 mg OD vs 150 mg OD dosing of enzalutamide where all patients (independent of dose) had exposure > 5 mg/L [37]. So for both erlotinib and enzalutamide it could be that patients are currently prescribed a higher dose than needed for maximal efficacy outcomes with a possible unnecessary increased risk for adverse events and also, additional costs.

When the DPOG-TDM study was opened, all oral targeted therapies which were prescribed and for which available validated assays were available were included. Additional research now has described the presence of a quantified exposure-response relationships as a necessary criterion for a drug to be suitable for TDM [2]. In addition to the previously described reasons for closing the palbociclib and olaparib cohort, the lacking evidence of a well quantified exposure-response relationship supported our decision [6]. If new evidence on these exposure-response relationships would become available, we would revisit our decision to close these cohorts. Since our study is dynamic, and not only can cohorts be closed, but also opened, we agreed that from now on we will only include drugs that have a well quantified exposure-response relationship at the standard dose, so the need and usefulness of TDM would be more obvious from the start [6]. Reflecting on the other closed cohorts, it is hereby important not to only have evidence for an exposure-response relationship, but also for an attainable and clear target. This is also our advice for other future research on routine TDM.

Although routine TDM was not feasible or useful for the described oral targeted therapies, TDM on indication could still be a useful tool in clinical decisions in individual cases. Patients using oral targeted therapies frequently use concomitant drugs that could influence exposure, such as Cytochrome P450 3A4 (CYP3A4) inducers and inhibitors. Sometimes these can be easily switched, but other times (e.g. patients with brain metastases with properly controlled epilepsy with anti-epileptic drugs) this is more difficult, and TDM could be used to provide information on exposure levels. TDM could also be useful in other situations that potentially influence drug exposure, such as gastro-intestinal surgery and the need for nasogastric tubes for the intake of food and drugs, and could be a tool to monitor patient drug adherence. For some drugs, it is known that patient specific characteristics as Body Mass Index can influence drug exposure, so TDM could be an useful tool to monitor exposure [38, 39]. If patients experience trAEs and need a dose reduction, TDM could also be helpful to titrate the dose to an acceptable and effective level [40]. Lastly, when patients experience AEs, high exposure could support the decision to lower the dose, because adequate exposure can still be guarded using TDM. In contrast, low exposure might support the decision to change treatment, because not only does the patient then experience AEs, also optimal efficacy might not be reached. Overall, drug exposure might be useful to take into account in clinical decisions.

## Conclusions

Therapeutic drug monitoring is a tool used to personalise treatment with oral targeted therapies. It has been proven valuable for many drugs, but we found some factors that influence the feasibility, usefulness and clinical applicability of TDM. Future research on routine TDM should focus on drugs with a well quantified exposure-response relationship and a clear exposure target, as these are crucial for the necessity of routine TDM. With this experience, we do not advise routine TDM to aim for an exposure target for cabozantinib, dabrafenib/trametinib, enzalutamide, erlotinib, everolimus, regorafenib and vismodegib, although TDM could still be used to guide clinical decisions in individual cases.

## Data Availability

The data that support the findings of this study are available from the corresponding author upon reasonable request.
